# Analytical performances of a novel point-of-care procalcitonin assay

**DOI:** 10.1016/j.plabm.2019.e00145

**Published:** 2019-10-26

**Authors:** Anne Marie Dupuy, Anne Sophie Bargnoux, Aneta Andreeva, Charlie Zins, Nils Kuster, Stéphanie Badiou, Jean Paul Cristol

**Affiliations:** a Laboratoire de Biochimie et Hormonologie, CHU Montpellier, Univ Montpellier 1, Montpellier, F-34295, cédex 5, France; b Laboratoire de Biochimie et Hormonologie, PhyMedExp, Université de Montpellier, INSERM, CNRS, CHU de Montpellier, France

**Keywords:** PCT, Correlation, Precision, Clinical concordance

## Abstract

**Objectives:**

We report the analytical performances of a new point-of-care (POC) procalcitonin (PCT) fluorescence immunoassay that uses the AFIAS-6© system from Boditech and its concordance with results of the standard method Kryptor Compact plus from the central laboratory.

**Design:**

and methods: Analytical performances including imprecision studies, limit of blank (LoB), limit of detection (LoD) and limit of quantification (LOQ) were determined. The method comparison was performed using plasma vs. whole blood for Kryptor CompactPlus© vs. AFIAS-6©, respectively.

**Results:**

The total imprecision was far from the CV of 4.5% claimed by the manufacturer and close to 10%, for levels of PCT at 0.4 and 8.3 μg/L. The LoD of this novel PCT assay was found to close to the LoD provided by the manufacturer at 0.04 μg/L. The LOQ was higher than that claimed by the manufacturer (0.1 vs 0.002, respectively). The equation of linearity in the lower range was found to be y = 1.056x – 0.039 with r^2^ = 0.993 with a mean recovery percentage of 86 ± 15%. Correlation studies showed a good correlation between PCT measurements using plasma on Kryptor system and on corresponding whole blood with POC reaching a bias of −0.04 in the range from 0.02 to 2 μg/L.

**Conclusion:**

The novel PCT assay on AFIAS-6© is an acceptable POC alternative for the diagnosis and management of sepsis at EDs to improve the flow of patients, as results are consistent with those of the standard PCT Kryptor Compact Plus© assay, despite its higher imprecision.

## 1 Introduction

There is a growing use of procalcitonin (PCT) measurements as a mean to diagnose and manage sepsis and PCT emerged as a useful tool to manage it accordingly, particularly in the emergency department (ED) and intensive care units (ICU). PCT has a fair diagnostic accuracy for bacteraemia in adult, neonates, infants, and children, hospitalized patients suspected of infection or sepsis. Low procalcitonin levels can be used to rule out the presence of bacteraemia. Usually, a PCT level≥0.5 ng/mL is considered to be positive for the diagnosis of a bacterial infection, for a PCT >2.0 ng/mL, a systemic infectious process is strongly suggested requiring a re-examination after 6 to 24 h even if bacterial infection or sepsis is suspected [1]. A level of >10 ng/mL indicates critical sepsis or septic shock. Rapid identification and management of systemic bacterial infections are fundamental in infants, and children as well as in adults. Indeed, a postponement in the treatment of severe bacterial infections may have a poor outcome. Distinguishing between a severe bacterial infection and a localized bacterial or a viral infection can be highly critical in treatment options [2].

Until recently, the only available methods for measuring PCT are the BRAHMS PCT Kryptor method. Other companies such as Abbott, Biomérieux, Diasorin, Fujirebio, Roche and Siemens by setting up a partnership with BRAHMS have also licensed the use of PCT and its antibodies. All commercial quantitative BRAHMS PCT assays use the same ‘sandwich ELISA’ principle to quantify PCT by forming antibody–procalcitonin–antibody complexes. The main difference between these assays is the mechanism of detection of these complexes. These assays have all been standardised using the original BRAHMS PCT Luminescent immunoassay (the original manual PCT assay). Recently, several companies have developed their own antibodies and have developed their own dosage. Three companies currently distinguish themselves from the rest in terms of their PCT detection products, including Radiometer with the AQT90 FLEX© PCT assay on AQT90 FLEX©, Diazyme laboratories with the Diazyme PCT assay that can be adapted for use with clinical chemistry analysers [3,4], and Boditech with its AFIAS© PCT Plus assay adapted on the new point-of-care (POC) AFIAS-6© system.

For these new assay methods not only the detection mechanism is different but also the antibodies. The latter, POC system was recently launched by Boditech adapted for emergency situations. The analytical performances and the perfect concordance of the POC system with the laboratory central method are necessary to use this POC with confidence and to allow relaying POC diagnostic in ICU to central laboratory method for follow up hospitalized patients. In this context, we report the analytical performances of the AFIAS© PCT Plus assay on the AFIAS-6© system and evaluate the concordance in results with the BRAHMS PCT Kryptor CompactPlus© system, considered as the reference method. Only the results of PCT concentration measured on plasma with KryptorCompactPlus© and on corresponding whole blood with AFIAS-6© will be discussed here.

## 2 Materials and methods

### 2.1 AFIAS© PCT plus method for PCT measurements

The AFIAS© PCT Plus assay was conducted using the Boditech AFIAS-6© instrument (Boditech Med Incorporated, Gang-won-do, Republic of Korea) distributed in France by Diasys’ society (Diasys, Montpellier, France). All reagents, calibrators, quality controls were manufactured by Boditech. The AFIAS© PCT Plus assay is a fluorescence immunoassay that quantifies PCT concentration in serum, plasma and whole blood (10–50 μL) drawn from a finger or heel using a capillary tip. AFIAS-6© POC uses an all-in-one cartridge which automates the entire procedure from sample preparation to test. Dried antibodies in a detector tube are diluted and bind antigens in samples to form antigen-antibody complexes which then migrate through a nitrocellulose matrix and are captured by another set of immobilised antibodies on the test area. Results were acquired after 12 min with a working range claimed by the manufacturer of 0.02–50 μg/L. Limit of blank (LoB), limit of detection (LoD) and limit of quantification (LoQ) were reported by the manufacturer to be 0.01, 0.02 and 0.02 μg/L, respectively.

### 2.2 BRAHMS PCT Kryptor CompactPlus© method for PCT measurements

PCT levels were determined using the BRAHMS PCT assay, which is routinely used at the central laboratory. The BRAHMS© PCT assay is an approach based on time-resolved amplified cryptate emission (TRACE) that is conducted using the Kryptor Compact Plus© instrument (B.R.A.H.M.S. AG, Hennigsdorf, Germany), anti-calcitonin polyclonal antibody conjugated with europium cryptate and anti-katacalcin monoclonal antibody conjugated with XL665. PCT measurements were obtained within 19 min on the Kryptor® system. The LoD and LoQ reported by the manufacturer were 0.02 μg/L and 0.06 μg/L, respectively.

### 2.3 Analytical performances of the AFIAS© PCT plus immunoassay

Imprecision studies were conducted in accordance to the CLSI EP15 protocol (five measurements per day on 3 levels for 5 consecutive days) [5] with heparinised plasma pools and control materials ready to use. The LoD was determined according to the current CLSI standards [6]. The LOQ was calculated according to the US FDA protocol [7] consisting in 5 times responses by blank. A heparinised plasma pool with a PCT concentration of 2.5 μg/L was used to test linearity in the lower range. The pool was diluted with diluent provided by manufacturer to the following final concentrations: 1.2, 0.8, 0.6, 0.3, 0.16, 0.08, and 0.04 and 0.02 μg/L. Each dilution was measured in duplicate.

### 2.4 Comparison studies

First, the comparison study between the AFIAS© PCT Plus and BRAHMS PCT Kryptor CompactPlus© assays was conducted using lithium-heparin samples from 118 consecutive patients admitted to the ED and the ICU departments of Lapeyronie university hospital (Montpellier, France) with values within the analytical range of 0.02–50 μg/L. When received, blood samples were split in two aliquots, one to be centrifuged for plasma PCT determination on the Kryptor CompactPlus, the other to be used simultaneously as whole blood on the POC system. No supplementary sampling was requested. The study was approved by local ethical committee. In the presence of discordant results between the two methods, the samples were re-analysed on the two instruments and the clinical record of patients were examined.

Secondly, to compare the POC assay against other methods, we measured on AFIAS-6© analyser external quality assessment (EQA) specimens stored at −80 °C from 2018 ProBioQual EQA program (ProBioQual, Lyon, France).

### 2.5 Statistical analysis

Passing-Bablok regression analysis was used to compare the results of the AFIAS© PCT Plus assay with the BRAHMS PCT Kryptor© assay. The scatter of differences was visualised by means of Bland-Altman plots [8]. The cusum test was used to detect deviation from expected values in linearity studies. Additionally, we took account of the concept of acceptable difference limit (ADL), which was calculated according to ISO 5725–6 [9]. Statistical analyses were performed using XLSTAT® software, version 2016.06.35661 (NY, USA).

The concordance between the two methods for classification of patients according to clinical algorithm was assessed using Cohen’s κ-test on the population divided into three categories: PCT values < 0.5; 0.5–1.99, ≥2.0 μg/L [10]. For all comparisons, *p*-value < 0.05 was considered statistically significant.

## 3 Results

Analytical performances (imprecision, LoQ, LoD, LoB and linearity study) of the AFIAS-6 © POC system are presented in Table 1. The total analytical imprecision was found to be close to 10%, which was twice higher than the coefficient of variation (CV) of 4.5% reported by Boditech. For higher concentrations of PCT, the AFIAS-6© exhibited an even high CV of 12%.

**Table 1:** Analytical performances of the AFIAS PCT assay on the Boditech AFIAS-6© analyser.

Total CV imprecision results	In our study			Manufacturer’ data	
	Mean, μg/L	CV, %		Mean, μg/L	CV, %
Plasma pool	0.4	9.9			
Control level 1	0.9	6.6	Control level A	0.21	4.6
Control level 2	8.3	12.2	Control level B	0.78	4.1
			Control level C	0.78	2.2

LoB	0.02			0.01	
LoD	0.04			0.02	
LoQ	0.1			0.02	

Linearity	
Theoretical values, μg/mL	Mean of observed values, μg/L (% of mean recovery)
1,25	1,34 (107)
0,80	0,75 (93)
0,60	0,58 (93)
0,30	0,25 (83)
0,15	0,11 (73)
0,08	0,05 (62)
0,04	0,03 (75)
0,02	0,02 (100)

CV: coefficient of variation; ND: not determined; LoB: limit of blank; LoD: limit of detection; LoQ: limit of quantification.

There was a gradual decrease in recovery from 107% on the highest sample (assigned value 1.25μg/L) to 62% on the low sample (assigned value 0.08μg/L). However, on the penultimate sample (assigned value 0.04μg/L) a positive bias was reported with a recovery of 75%.

The AFIAS-6© PCT Plus assay displayed acceptable linearity over the most clinically relevant range for PCT concentrations with a trend of gradual decrease in recovery. The Passing-Bablok regression showed a break in the dilution series at the penultimate sample (0.04 μg/L). However, the Cusum test did not show significant deviation from linearity.

**Fig. 1:**
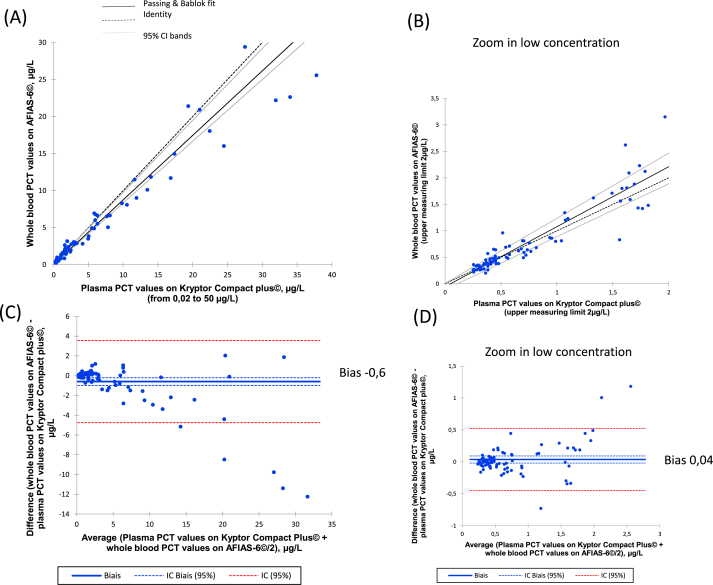
(A) Passing-Bablok regression analysis of plasma samples with the Kryptor© system versus whole blood samples with the AFIAS-6© system in the range 0.02–50 μg/L, and (B) in the low concentration from 0,02 to 2 μg/L. The vertical and horizontal solid lines represent the 0.5 μg/L threshold, while the dotted lines represent the 95% confidence limits. (C) And (D) Bland-Altman analysis for measurements of plasma samples using the Kryptor© system versus whole blood samples using the AFIAS-6© system.

The regression analyses between the PCT measurements of 118 plasma samples on the Kryptor Compact Plus© and corresponding whole blood samples on the AFIAS-6© analyser are reported in Fig. 1. The mean difference in PCT measurements for the entire measurement range was 4.3% (SD = 22%). For PCT values < 0.5, between 0.5 and 2 and ≥ 2 μg/L, the mean percentage of over- or under-estimation was −0.8, −1.0, 13%, respectively. We observed a trend to underestimate low PCT values (0.2 to 2 μg/L) and overestimate PCT values > 2 μg/L with the AFIAS-6© method. However, the PCT assay on AFIAS-6© exhibited good correlation in the working range of 0.02–50 μg/L and an acceptable concordance with a bias of 0.60 (±2.11) for whole blood sample measurements when compared with plasma sample measurements of the Kryptor Compact Plus© method.

We focused on the low PCT values comprised between 0.02 to 2 μg/L because AFIAS-6© system should be considered as a screening tool using the cut-off 0.5 μg/L. The agreement in PCT measurements was excellent between the AFIAS-6© and Kryptor Compact Plus© systems as indicated by the correlation coefficient >0.92 (Fig. 1A). At the threshold of 0.5 μg/l, three PCT measurements were inconsistent between the two methods (1 patient with sepsis, and 1 patient with leukemia, 1 liver transplant patient. However, these three measurements remain close around 0.5 μg/L (sample 1: 0.43 vs.0.65 μg/L, sample 2: 0.45 vs 0.64 μg/L and sample 3: 0.70 vs.0.49 μg/L as reported by the Kryptor© Compact Plus vs. the AFIAS-6© system). Overall, the discrepancies in values measured by these methods would have few impact on the clinical care provided to patients. Bland Altman analysis further confirmed this good agreement (Fig. 1B) with a bias of −0.04 (±0.24). We calculated the ADL threshold to be 41% and found six inconsistent results between plasma sample and whole blood sample measurements. Thus, PCT measurements with the Kryptor© Compact Plus and AFIAS-6© systems were classified as not being significantly different.

The Kappa coefficients were also determined to be close to 0.84 and consequently, the strength of agreement is considered to be very good between the two methods. Hence, the differences in these methods are minimal and the use of the AFIAS© PCT POC in ED would be suitable for bacterial infection diagnosis.

Levels of EQA samples obtained from the ProBioQual registry, using different methods, including the Kryptor© Compact Plus system (Thermofisher), were compared with PCT measurement on the AFIAS-6© system (see Figure A in supplementary appendix).

For PCT concentrations between 0.2 to 10μg/L, the AFIAS-6© system results were higher than those Kryptor© Compact Plus instrument but lower than those obtaiend with other systems, particularly for values greater than 6 μg/L. The differences in PCT values that were obtained with the AFIAS-6© system and those generated by other groups could be due to matrix effects of EQA samples or differences in measurement methods.

Taking into considerations these results, it could be assumed that AFIAS-6© PCT is a new system, with results nearer to Kryptor© Compact Plus instrument than other systems (Biomérieux, Radiometer, Roche, Siemens). »

## 4 Discussion

Analytical performances of the AFIAS-6 © POC system were acceptable to use in triage process at ED. Since a few years, EDs congestion is worsening demanding. Factors that impact patient flow into EDs were now well recognised. Increases in the number of ED presentations, increasing aging populations, increasing incidence of chronic conditions and difficulty accessing primary/general practice and community services, all increase healthcare demand and the flow of patients into EDs [11]. In consequence, the establishment of a POC system at EDs could have an impact on the improvement of the flow of patients with a decreasing length of stay in the ED. However, the obtained CVs ranged from 6.6 to 12.2%, were higher compared to those claimed by the manufacturer and those found with other POC PCT systems, such as AQT90 Flex © (CV of 3.9%) [12]. These observations were in line with the underestimation of low PCT values and the trend of overestimation of high PCT values that we found for the AFIAS-6© system in our comparison studies. These data on reproducibility are important especially when PCT-based algorithms are implemented for antimicrobial stewardship purposes. In this case, strict cut-off values are commonly used to decide start or discontinuation of antibiotic treatment [13]. In addition, strict cut-off values are difficult to consider because of the lack of reference material for the PCT to demonstrate the accuracy of measurements and methodologies. In view of our results for the AFIAS-6 © POC system, this POC system, did not seem to be indicated for the initiation or escalation of antibiotic therapy. However, this level of imprecision remains acceptable according to the recommendations of Jaffe et al. [14], which states that assays with an imprecision up to a 20% CV may reasonably usable and acceptable for a POC system. Thus the final recommendations, could be around the use for diagnosis and non use for the management of antibiotic therapy. In addition, the difference across all the methods as seen with EQA samples, highlighted a lack of standardisation of the different PCT assays.

## 5 Conclusion

In summary, we conclude that the novel AFIAS-6© system-based POC PCT assay is an acceptable and suitable POC method for the diagnosis of bacterial infection and sepsis at Emergency department. Due to its relatively poor precision, the POC system is less recommended to manage antibiotic therapy. The ability to use whole blood samples and its usable by medical personnel make this method very appropriate in a clinical setting such as ED.

## Declaration of competing interest

The authors declare that the research was conducted in the absence of any commercial or financial relationships that could be construed as a potential conflict of interest.
