# Supplementary material for: Analytical performances of a novel point-of-care procalcitonin assay
Source: Pract Lab Med. 2019 Oct 26;18:e00145. doi: 10.1016/j.plabm.2019.e00145 (PMC6838538; doi:10.1016/j.plabm.2019.e00145)
Supplement: Multimedia component 1 [file mmc1.docx]

**Supplemental Figure A:** Comparison of PCT measurements on ProBioQual samples generated by the AFIAS-6© analyser and other PCT measurement methods. PCT levels were measured using the Vidas© system from Biomérieux, AQT90flex© system from Radiometer, Cobas© system from Roche, Advia Centaur© system from Siemens and Kryptor© compact plus system from Thermofisher. EQA samples had PCT concentrations ranging from from 0.2 to 10 µg/L.
